# Assessing the implementation of the disease surveillance and response strategy in Togo

**DOI:** 10.1371/journal.pgph.0005698

**Published:** 2026-09-09

**Authors:** Dibeka Baman, Stéphanie Maltais, Laurent Cleenewerck de Kiev, Komla Kadzahlo

**Affiliations:** 1 Division of Public Health Institutes and Research, Africa CDC, Addis Ababa, Ethiopia; 2 Department of Global Health and Health Systems, Euclid University, Bangui, Central African Republic; 3 Department of Health Management, Evaluation and Policy, School of Public Health, Université de Montréal, Montréal, Canada; 4 Centre de recherche en santé publique (CReSP), University of Montreal and CIUSSS du Centre-Sud-de-l’Île-de-Montréal, Montréal, Canada; 5 Department of Malaria Prevention, Ministry of Health, Lomé, Togo; SUPA71 Co., Ltd, THAILAND

## Abstract

The surveillance of infectious diseases has gained importance with the rise of (re)emerging pathogens and growing antimicrobial resistance. In many low- and middle-income countries (LMICs), surveillance systems remain insufficiently structured to support timely detection and response. To address these gaps, the World Health Organization Regional Office for Africa recommends the Integrated Disease Surveillance and Response (IDSR) strategy, implemented across 47 Member States. Regular evaluation of IDSR systems is encouraged to guide national reforms and strengthen preparedness. This study assesses the level of IDSR implementation in Togo and identifies key challenges affecting its performance. A qualitative study was conducted through a review of national strategic documents and focus group discussions with 21 actors involved in IDSR, including national surveillance specialists, Public Health Emergency Operations Center staff, decentralized health authorities, health facility managers, community health workers, and community leaders. Data were collected using tailored interview guides and analyzed through content analysis structured around the nine components of the WHO IDSR Technical Guide. IDSR is only partially implemented in Togo. While some opportunities and technological tools are in place, major weaknesses in the health system limit the effectiveness of surveillance. Significant gaps remain in coordination, resource mobilization, health system effectiveness, data quality and reporting, preparedness, and response capacities. Significant gaps hinder the full implementation of the IDSR in Togo. Addressing these challenges presents opportunities to strengthen surveillance, improve early detection, and enhance public health response across the country.

## Introduction

Regardless of their geographic origin, infectious and communicable diseases pose significant public health and biosecurity threats to all countries. To minimize the risks associated with such trade, the Codex Alimentarius Commission and the *Office International des Epizooties (OIE)* have established sanitary rules to mitigate these risks [1–4]. Integrated Disease Surveillance and Response (IDSR) serve as the primary operational framework through which countries develop, strengthen, and sustain the surveillance, laboratory, reporting, preparedness, and response capacities required under the IHR (2005), thereby enabling the early detection, assessment, notification, and containment of public health threats before they escalate into national, regional, or global health emergencies [1,3]. Accurate and up-to-date knowledge of the epidemiological status of critical infectious and transmissible diseases has become increasingly indispensable, [1,5,6] if not mandatory. International trade regulations require countries to provide scientific evidence before imposing restrictions on international trade [1,7]. Furthermore, epidemiological surveillance is essential for protecting populations from new or exotic diseases and implementing and evaluating control programs. Recently, there has been a significant increase in consumer concerns about food safety, and the medical profession is eager to receive warnings of potential health issues [1,8]. Both human and animal epidemiological surveillance have an increasingly important role as epidemiological data have become the core of practical global cooperation on trade and health.

The overarching goal of infectious disease surveillance is to minimize their impact by providing decision-makers with useful information, enabling them to plan appropriate control and intervention measures, allocate sufficient health resources, and make informed case management recommendations [9]. Epidemiological surveillance is defined as a system based on continuous information recording to monitor the health status of a given population and the risk factors to which it is exposed, to detect pathological processes as early as possible, and study their development in time and space, to take appropriate measures to control them [1,10]. Emerging zoonotic diseases have had dramatic and devastating effects globally, especially in LMICs [11,12].

In 1998, in response to an increasing number of (re) emerging diseases causing high mortality and morbidity in Africa, the WHO Regional Committee for Africa adopted Integrated Disease Surveillance to create and implement comprehensive, action-oriented, integrated, and district-focused public health surveillance for African countries [13,14]. The aim is to address fragmentation caused by multiple vertical, disease-specific surveillance programs and to improve early detection and timely response to public health issues [15,16].

The primary objective is to build a functional, integrated, and sustainable national surveillance system to enable early detection of epidemic-prone diseases, prompt reporting and laboratory confirmation, timely and coordinated public health response, monitoring of disease trends for informed decision-making, and strengthening overall health system resilience [15–17]. Moreover, this strategy, by supporting the implementation of the International Health Regulations (IHR 2005), contributes to global health security [17]. It is built on several key principles, such as integration, an action-oriented approach, decentralization, capacity building, standardization, and community involvement [17,18].

The strategy defines essential core surveillance functions, which include: case detection, registration and confirmation, reporting, data analysis and interpretation, feedback, preparedness, and response [17,18]. Furthermore, to ensure effective implementation, IDSR emphasizes several support functions: training and workforce development; laboratory strengthening; supervision and mentorship; logistics and supply systems; coordination mechanisms; and monitoring and evaluation [16,19].

Countries adapt IDSR to their epidemiological context. However, it generally includes epidemic-prone diseases, diseases targeted for elimination or eradication, high-burden endemic diseases, emerging and re-emerging infections, and public health events of international concern [15,17]. Effective implementation of IDSR is expected to result in improved timeliness and completeness of reporting, faster outbreak detection and response, reduced morbidity and mortality from epidemic-prone diseases, strengthened national compliance with IHR (2005), and enhanced health system resilience [15,17,18].

In Togo, while routine performance reports and periodic joint external evaluations (JEE) provide quantitative indicators of surveillance performance for rapid decision-making, fewer studies have explored the implementation of the IDSR strategy in practice, particularly from the perspectives of frontline health workers and decision-makers. A better understanding of operational realities, resource constraints, governance dynamics, and contextual facilitators is essential to strengthen implementation and inform policy reform.

The aim is to assess the implementation of the IDSR strategy in Togo using documentary analysis and focus group discussions. More specifically, we seek to examine the alignment of national policies, guidelines, and operational plans with IDSR core and support functions, identify gaps between policy intent and field-level implementation, and explore stakeholder perceptions of strengths, challenges, and opportunities for improvement.

### Methodology

#### Ethics statement.

The study was approved by the Health Research Ethics Committee of Togo and authorized by the Ministry of Health (N° 325/2024/MHPH/GS/GDRPHI/DRPP/RSD). Written informed consent had been obtained from all FGD participants, and confidentiality and anonymity were ensured by removing every identifying information from transcripts and reports.

#### Study design.

A qualitative descriptive design was employed, combining documentary analysis and focus group discussions (FGDs). The main question this study intended to answer was: How far has the implementation of the IDSR strategy progressed in the Togolese Republic? This approach enabled triangulation between policy-level documentation and experiential insights from key stakeholders involved in implementing the IDSR strategy.

#### Study setting.

The study was conducted in Togo in December 2024 at the national, regional, and district levels of the health system, which is structured into central, intermediate, and peripheral levels, with surveillance activities implemented across public and private health facilities. The research participants were recruited from December 5, 2024, through December 25, 2024

#### Documentary analysis.

We used a purposive sampling strategy to identify the most relevant documents produced between 2010 and 2025. These strategic documents include national surveillance policies and strategic plans; technical guidelines and training manuals; standard operating procedures (SOPs); outbreak investigation reports; annual health statistics reports; monitoring and evaluation frameworks; and technical reports from partners on surveillance. The documents were obtained from publicly accessible institutional archives and the Ministry of Health.

We used a structured data extraction grid, developed from the WHO IDSR framework, which mainly includes core functions (case detection, registration, confirmation, notification, analysis, feedback, preparedness, response), support functions (training, supervision, laboratory capacity, logistics, coordination, monitoring and evaluation), and system attributes (timeliness, completeness, data quality, flexibility, acceptability, stability). The content analysis was intended to grasp the theoretical and practical aspects of IDSR implementation in Togo and to confront them. Documents such as surveillance protocols, IDSR guidelines, and procedures were essential for a better understanding of theoretical elements. By contrast, documents like annual reports and Joint External Evaluation (JEE) documents were critical in assessing practical aspects.

Content analysis was conducted using a thematic framework, and documents were coded deductively according to the domains of the Integrated Disease Surveillance System (IDSR). However, these documents were coded inductively for emerging themes related to intersectoral collaboration, governance, financing, and digitalization.

#### Focus group discussions.

Focus groups for this study were organized with key stakeholders in disease surveillance. Specifically, we focused on participants such as district-level surveillance officers, health facility liaisons, laboratory staff, regional health managers, private facility managers, and community health representatives. Participants were chosen to ensure representation from different geographical areas and levels of the health system. The first FGD lasted three hours and brought together three Specialists from the Directorate of Surveillance and Intelligence in Emergency and Response Health (DSIERH) who had been with the Directorate for at least five years. The second one lasted about 2 hours and brought together 3 specialists from the Public Health Emergency Operations Center (PHEOC), with a combined experience of at least 5 years. The third FGD, which lasted two hours, brought together decentralized representatives of the Ministry of Health (2), health facility managers (2), and the disease surveillance focal point. The last FGD comprised community health workers (5) with at least 5 years of experience and community leaders (5) and lasted about 4 hours. A total of 21 respondents participated in the research

For data collection, a semi-structured interview guide was developed and utilized to gather participants’ perceptions and impressions of the system’s strengths, weaknesses, and opportunities for improvement; the adequacy of training and supervision; data management and feedback mechanisms; coordination during outbreaks; and resource availability; and their experiences with surveillance, reporting, and response. These focus group interviews were conducted in French, recorded with participants’ consent, and fully transcribed.

For data analysis, the transcripts were analyzed using thematic content analysis. Coding was conducted manually in Excel by two researchers following brief training to ensure a shared understanding of the coding framework and analytical approach. Coding discrepancies were resolved by consensus. The involvement of two analysts aimed to enhance the credibility and reliability of the findings through investigator triangulation. The analysis involved two rounds of coding. In the first round, two researchers independently conducted deductive coding based on the WHO IDSR framework while remaining open to inductively emerging themes. In the second round, the researchers jointly reviewed and refined the coding framework, discussed coding discrepancies, merged overlapping codes, and reached consensus on the final themes. In validation procedures, selected participants were invited to review the interpretation of the findings to ensure that the themes accurately reflected their experiences, including the inductively derived themes. We used a hybrid deductive and inductive approach (deductive coding aligned with the components of the IDSR framework, while inductive coding aimed to capture contextual and operational realities in practice in the country). Because the study employed purposive sampling to capture perspectives across the principal levels of the health system rather than to generate theory, we assessed thematic saturation pragmatically. No substantially new themes emerged during the fourth focus group discussion, and additional data were considered unlikely to change the analytical framework. Triangulation was performed using documentary and focus group data to strengthen the credibility of the results. Discrepancies between policies and practices were systematically identified.

Data triangulation, based on policy documents and stakeholder perspectives, was implemented, and validation was conducted with selected participants. In addition, a peer debriefing was held within the research team, and a written record of coding decisions was maintained. When discrepancies arose between documentary evidence and focus group findings, these were not treated as inconsistencies to be resolved but as analytically meaningful findings. Therefore, each discrepancy was examined by returning to the original documents and transcripts, discussed among the research team until consensus was reached, and reported as evidence of gaps between policy intentions and implementation in practice.

## Results

As IDSR involves all levels of the health system in the surveillance and response to priority diseases and conditions [20], the following sections examine how the IDSR’s core components are structured and implemented in the Togolese context.

### Identify and record cases of illnesses, conditions, and events

In Togo, the IDSR strategy includes indicator-based and event-based surveillance to rapidly detect priority diseases, conditions, and events, as stressed by one participant, “An effective epidemic intelligence system contains both indicator-based and event-based surveillance.” Human, animal, and environmental health personnel conduct surveillance activities at the peripheral, intermediate, and central levels of the health system to identify health issues of concern to the community. The participants unanimously recognize the critical roles of community members in epidemiological surveillance, “as they facilitate the early detection of priority diseases, events, and conditions and take appropriate action”. In Togo, they are oriented and trained to actively participate in detecting, reporting, responding to, and monitoring human or animal health events in their intervention area, based on documentary evidence. According to most participants, “personnel at the border (points of entry) are also trained to identify and notify of these events, facilitating a prompt response”. The focus is on detecting not only known public health threats using established case definitions and official reporting pathways, but also events or risks not expressly included in the official reporting system. However, most study participants disagree with this claim. They believe their training is insufficient and somewhat inappropriate, and that the staff’s proficiency falls below expectations. The IDSR strategy utilizes indicator- and event-based surveillance to detect diseases, conditions, and events, thereby enhancing surveillance sensitivity across all levels of the health system. Indicator-based surveillance uses standard case definitions to identify diseases, conditions, and events, whereas event-based surveillance uses detection, triage, and alert verification to identify specific events. Unlike case definitions that are narrow and disease-specific, event surveillance requires detecting and immediately reporting broad alerts that signal the possibility of a serious public health event. “We have to think out of the box”, stressed one participant. Based on standard case definitions, a three-tier classification system is typically used at the health facility level: suspected, probable, and confirmed. Moreover, to comply with the One Health approach, a holistic approach is applied to joint event detection and risk assessment of potential public health events occurring at the interface between humans, animals, and the environment. The description of the geographical area served, the list of reporting sites, the names of surveillance focal points in the health district, and the names of community representatives involved in community monitoring are regularly updated.

The main challenge recognized by most participants was the lack of a laboratory quality control and assurance program. It was also recognized as a shortcoming in multiple strategic documents. Togo faces significant challenges in human and financial resources for Integrated Disease Surveillance and Response (IDSR), marked in particular by a shortage and an unequal distribution of qualified personnel in epidemiological surveillance. This shortage significantly limits the effectiveness of identification, according to participants.

After identifying and recording the case, notifying the relevant authorities of priority illnesses is the next step. However, data on the completeness of reporting, the percentage of expected reports actually received within the reporting period, is unavailable even in the annual reports.

### Notifying priority illnesses, conditions, and events

As a State Party to the International Health Regulations 2005 (IHR-2005), Togo adheres to the required protocols, whereby its system mandates the immediate reporting of diseases with epidemic potential or other Public Health Emergencies of International Concern (PHEIC). “When such an event is identified within a community, health facility, or at a point of entry, it must be promptly communicated to the national IHR focal point and the designated regional monitoring authority”, stressed one participant. Table 1 lists the infectious diseases that require immediate notification in the Togolese Republic. Immediate reporting of information, such as single cases or groups of notifiable events, generates an alert and initiates the case reporting system. Specific details on the suspected case, or if it involves a group of cases, are collected in full and passed to the next level. For events notified at the point of entry, the information is communicated to the health district where the point of entry is located, and simultaneously to the national IHR coordinator. The majority of the participants reject this claim, pointing out some logistical issues that hamper immediate notification in some circumstances. The Summary of diseases, conditions, and events requiring immediate notification is analyzed and monitored on a weekly, monthly, and triannual basis.

**Table 1:** List of infectious diseases requiring immediate notification in Togo.

Infectious diseases requiring immediate notification in Togo		
1. Ebola virus disease	11. Dracunculiasis	21. Rage (human)
2. Marburg virus disease	12. Influenza (new subtype)	22. Smallpox
3. Lassa fever	13. Measles	23. Noma
4. Rift Valley fever	14. Monkeypox	24. Bruruli ulcer
5. Crimean-Congo fever	15. Plague	25. Lymphatic filariasis
6. Carbuncle	16. Trachoma	26. African Trypanosomiasis (HAT)
7. Bacterial meningitis	17. Onchocerciasis	27. Yaws, endemic syphilis
8. Cholera	18. Neonatal tetanus	28. Yellow fever
9. Severe diarrhea	19. Leprosy	29. Zika virus disease
10. Shigellosis	20. Poliomyelitis	30. Covid-19/SARS

Source: Ministère de la santé et de l’Hygiène publique. “Surveillance intégrée de la maladie et riposte: Guide technique.” MSHP, 2022. https://sante.gouv.tg/documentation.

However, some participants noted a weak link between laboratory and surveillance data. It is crucial to highlight problems in communication and information exchange, as well as the lack of clear delineation of roles and responsibilities within the IDSR information transmission mechanism. These difficulties are thought to be particularly acute in laboratories responsible for case confirmation, underscoring the need for a structured, coherent approach to overcome them. Moreover, participants highlight the need for deeper integration with the National Health Information System (SNIS) to address shortcomings stemming from the lack of reporting by many facilities, especially private ones. Also, “the timeliness of reporting needs to be calculated each year to evaluate the IDSR implementation”.

IDSR is a system designed to ensure the transmission of reliable epidemiological information at the national level and to meet the requirements of the International Health Regulations. According to the Togolese system, it is essential to provide surveillance data reporting to all stakeholders who need this information to identify problems, emerging conditions, and priority health events, and organize the appropriate response by informing the actors involved in surveillance at all levels and on a sectoral basis. It is also critical to take timely action, monitor the evolution of diseases, conditions, and health events in the region, and assess the effectiveness of the response.

### Analyzing and interpreting data

Data analysis and interpretation are crucial for observing trends over time, alerting health personnel and relevant stakeholders, identifying areas of higher risk, describing individual variables, and monitoring and evaluating public health interventions. The country system monitors data flows from reporting sites to the central level. The information collected on the site is compiled on standard sheets, analyzed, and then transmitted to the health district’s team management. In settings with established electronic surveillance systems, data are entered via mobile phones or computers, allowing health district management teams and higher administrative levels to access the compiled information directly from their computers. Health districts merge, aggregate, and transmit their data and reports to the regions, then to the areas, and finally to the central-level health authorities. At all levels where data is received, the surveillance team liaises with the manager in charge of health information management to extract the data on IDSR priority diseases and record them on the IDSR summary sheets from all reporting sites. “Data are analyzed based on time, location, and individual characteristics,” according to participants. Also, data quality (consistency between registers and reports), and coverage of reporting sites are measured. However, there is no data to confirm this assertion.

The lack of commitment, especially from private facilities, renders data analysis and interpretation challenging. Another challenge pointed out by participants was the lack of collaboration between surveillance focal points and laboratories. This lack of collaboration, according to them, often leads to delays in data entry, thus affecting data transmission and analysis.

### Investigating suspected outbreaks and other public health events

The Togolese system considers an epidemic as a sudden increase in the number of cases of a disease, or the occurrence of an event beyond what is usually expected within a population in a given geographical area and during a given period. In Togo, the preparedness of the investigation aims to mobilize the Rapid Intervention Team (RIT) in a public health emergency, specify the respective tasks and roles incumbent on the members of the RIT, define supervision procedures and communication strategy, determine the location(s) of the investigation, obtain the necessary authorizations, develop forms and methods for collecting information and taking samples, organize travel and other logistical aspects, and gather the indispensable equipment for taking samples for laboratory analysis. This involves providing the intervention team with “appropriate information and data on the suspected illness to guide the investigation and determine the necessary precautions”. Clinical and epidemiological history and biological sample analysis are used to verify and confirm an outbreak or public health event. Defining and searching for additional cases, reporting and communicating survey results, implementing measures to prevent and contain epidemics, carrying out an assessment to determine whether the event constitutes a potential public health emergency of international concern, maintaining and strengthening surveillance, and carrying out a regular risk assessment after the outbreak is confirmed are the essential other activities during an investigation of a suspected outbreak.

The main challenge, as cited by most participants, was implementing measures to prevent and contain epidemics while investigating. According to them, mobilizing the RIT and equipping them with clear, pre-determined criteria is not enough for the investigation process to be successful. There is no data on the case detection and confirmation rates for priority diseases, though this is essential, alongside specimen referral turnaround time.

### Preparing to respond to epidemics and other public health events

Preparing for and responding to public health events in Togo involves establishing a committee to manage epidemics and other public health emergencies at the district and regional levels, known as the Health Emergencies Management Committee (HEMC). At the national level, the National Institute for the Coordination of Disease Surveillance and Control (NICDSC) and the Public Health Emergency Operations Center (PHEOC) serve as a Command-and-Control center for coordinating emergencies and public health events. Policies, plans, and procedures for conducting operations are developed to take inventory of available resources, estimate requirements, build up reserve stocks, and organize simulation exercises to test systems. Key members of the epidemic and other public health event management committees, as well as rapid intervention teams, are identified and trained in advance to respond promptly to potential emergencies according to documentary analysis. Vulnerability, assessment, and risk mapping are used as preparedness aids to identify areas or populations at risk, classify preparedness activities, and engage with key political and operational partners.

However, participants agreed that training in responding to epidemics was insufficient. According to most of them, integrating IDSR modules into the initial training of healthcare professionals and developing specific modules on diseases with epidemic potential could be leveraged. “It would be beneficial to update the current staff’s knowledge and skills and familiarize them with innovative surveillance tools, such as EIOS.”, stressed one participant. One can note a discrepancy between theory and practice. Moreover, evaluating outbreak detection, response time, and the availability of rapid response teams is challenging, as there is no data available on these metrics.

### Responding to outbreaks and other public health events

In Togo, once an epidemic threshold is reached at the district level, the Health District Director informs the region and the central level. At the central level, depending on the scale of the event, the IDSR division and the IHR National Focal Point determine, in accordance with the IHR decision instrument, whether it is a potential public health event of international concern (PHEIC). Response activities in the event of an epidemic, emergency, or public health event usually consist of strengthening case management and infection control measures, improving the skills of response personnel, strengthening surveillance during response activities, including with neighboring districts, engaging the community in response activities, informing and educating the community, conducting a mass vaccination campaign, improving access to clean drinking water, ensuring the elimination of potentially infectious waste, improving food handling practices, reducing exposure to contagious or environmental risks, ensuring the handling and burial of corpses in a dignified and secure manner, and guaranteeing appropriate and adequate logistics and supply. Submitting regular situation reports on epidemics and other events, and documenting the response, are also essential.

However, responding to outbreaks and other public health events in Togo faces multiple challenges. The delay in reporting results and the lack of fluidity in collaboration were the most cited. Moreover, the fragmentation and weak integration of the various surveillance and response programs and actors were recognized as leading to duplication of effort and to reduced effectiveness across the different phases of the integrated management of Risks. Furthermore, assessing training coverage of health staff in IDSR is challenging, as there is no data on, availability of standard guidelines/tools, and functionality of community-based surveillance.

### Communicating about risks

In Togo, communication targets the community, surveillance agents (human, animal, and environmental health; laboratory), public and private health facility providers, personnel working in the platforms of land, air, and port entry points (travelers, immigration agents, airline staff, carriers, etc.), decision-makers, media professionals as a channel to reach these audiences, schools and workplaces and religious and traditional authorities. Communications leaders take advantage of the absence of an emergency to strengthen national communications capacity and develop communications plans and tools to enable the country to achieve a high level of communications preparedness. During the epidemic, the main activities consist of identifying and coordinating partners and other stakeholders, communicating with affected communities and stakeholders, distributing IEC materials and developing summary sheets, developing and disseminating public health situation reports, and communicating with the media and health workers. The Public Health Emergency Management Committee, through the Public Health Emergency Operations Center, ensures that communications are consistent with data analyses and that the objectives of communication activities are transparent and precise, taking into account the community’s experiences and expectations regarding the epidemic. Following the epidemic, a response report is prepared, and the lessons-learned evaluation aims to strengthen effective public responses to similar emergencies in the future. The country recently began piloting the platform “Epidemic Intelligence from Open Sources” (EIOS). However, most participants recognized that it could be challenging to expand it nationwide. Moreover, regular bulletins constitute feedback mechanisms integrating with DHIS2.

### Monitoring, evaluating, supervising, and reporting information to improve surveillance and response

In Togo, regular evaluation of information allows supervision team members to determine whether the surveillance and response objectives have been achieved and whether the results are of the expected quality. Supervision from the health ministry encourages healthcare professionals to work together to review progress, identify problems, determine their causes, and propose concrete solutions. The identification and use of indicators prove effective in measuring the degree of achievement of a particular program or activity. “Core function indicators assess the processes and achievements of the surveillance system”. In the Togolese system, monitoring the essential functions of the IDSR at the health district level involves identifying /detecting cases and public health events, notifying cases and events, analyzing and interpreting data, investigating and confirming suspected cases or outbreaks, preparing and responding to outbreaks, sharing feedback, and ensuring the reliability of the system.

Facilitative supervision is recognized as a process that promotes the quality of care and services at all levels of the health system by strengthening relationships within it, focusing on the identification and resolution of problems, optimizing the allocation and use of resources, promoting norms and standards, fostering teamwork, and facilitating bidirectional communication. Supervisors and healthcare professionals review progress in the Togolese system, identify problems, determine their causes, and propose concrete solutions. The effectiveness of the IDSR system’s implementation is ensured by setting objectives, selecting appropriate indicators, developing evaluation methods and tools, and designating those responsible for data compilation, organization, and analysis. Moreover, successful activities are integrated into the health district plan, and the applicable solutions are chosen following the annual assessment review. The results of the evaluation activity are also reported and shared with health centers and other stakeholders in the health district. However, an epidemic intelligence system that encompasses early warning functions for human and animal infectious diseases is to be developed. Moreover, participants recognize the lack of benchmarking against Joint External Evaluation (JEE) scores measurements and WHO IDSR technical guidelines enables regional comparison. Annual reports do not systematically include core indicators, such as completeness and timeliness of reporting, data quality (consistency between registers and reports), coverage of reporting sites, case detection and confirmation rates for priority diseases, alongside specimen referral turnaround time. Also, evaluating outbreak detection and response time and the availability of rapid response teams is not systematical. This is recognized as the system gap by participants.

### Using the electronic integrated disease surveillance and response system (e-IDSR)

Electronic tools in the health sector can produce validated data in real-time for public health surveillance, investigations, and rapid response to epidemics. Participants were almost unanimous on that. Consequently, they believe that electronic IDSR offers new opportunities to accelerate the realization of the basic capabilities outlined in the IHR (2005). However, the remaining participants were reluctant, citing technical challenges.

The Togolese system relies on health information management systems to facilitate systematic data collection, which supports planning, management, and decision-making in health services. These systems systematically collect data on illnesses, events, conditions, and other administrative and service delivery data. The first source of this data is the register of outpatient consultations or hospitalizations of the health facility. “Although it is challenging, efforts are underway to implement the District Health Information System version 2 (DHIS2) nationwide”, stressed some participants. The National Health Information System (NHIS) and the electronic IDSR draw data from outpatient consultations and hospitalization registers. However, there are no long-term strategic plans and frameworks for e-health in countries, including health and medical IT, telemedicine, E-learning, and mHealth.

### Togolese context-specific challenges falling outside the technical scope of the WHO’s IDSR core functions

Multiple context-specific challenges fall outside the technical scope of WHO’s IDSR core functions in Togo. Weak enforcement of reporting obligations in private and faith-based facilities, fragmented institutional mandates across central and peripheral levels, and limited accountability mechanisms for data quality and timeliness are the main emerging governance constraints. Private facilities are not accountable for their actions toward IDSR’s success, leading to a lack of commitment. This situation leads to frustration among public staff, according to a participant. Moreover, reliance on donor-driven vertical programs and frequent turnover of district leadership further undermines policy continuity and national ownership. According to some participants, each partner conducts its program according to its vision and objective, which do not always comply with the national priority. The system is overly dependent on external partners, creating parallel coordination structures that dilute the Ministry’s authority.

Across human, animal, environmental, and other sectors, intersectoral collaboration remains ad hoc despite One Health commitments. Participants think that data-sharing protocols across diverse ministries are poorly formalized, and joint outbreak investigations are inconsistently institutionalized. “More efforts need to be made”, stressed one participant. Furthermore, limited engagement of local authorities and civil society significantly reduces trust and event-based reporting. Also, most participants believe that cross-border coordination with neighboring countries is operational but not sufficiently harmonized, reflecting broader governance and regional integration constraints.

Table 2 summarizes the system’s key results and shortcomings/challenges.

**Table 2:** Key results and shortcomings/challenges of the system.

Steps	Results	Shortcomings/Challenges
Identify and Record Cases of Illnesses, Conditions, and Events	Existing programs for Stakeholders’ orientation and training to actively participate in detecting, reporting, responding, and monitoring human or animal health events in their intervention area.	Establish a laboratory quality control and assurance program.
Notifying priority illnesses, conditions, and events	-List of infectious diseases requiring immediate notification -Togo adheres to the IHR protocols	-Strengthening links between laboratory data and surveillance data -Promoting a multi-sectoral “One Health” approach
Analyzing and interpreting data	-Standard sheets used to collect, compile, and analyze data -The use of mobile phones or computers	Poor use of the electronic system in rural areas
Investigate suspected outbreaks and other public health events	-Mobilization of the rapid RIT -Clear pre-determined criteria	Implement measures to prevent and contain epidemics.
Prepare to respond to epidemics and other public health events	-HEMC at the district and regional level -NICDSC at the national level -PHEOC	Strengthen case management and infection control measures. Insufficient domestic resources
Responding to outbreaks and other public health events	The implementation of the procedure for each PHEIC is coordinated at the central level	The delay in reporting results and the lack of fluidity in collaboration
Communicate about risks	Risk communication is coordinated by the Public Health Emergency Management Committee through the Public Health Emergency Operations Center, ensuring that communications are consistent with data analyses and that the objectives of communication activities are transparent and precise. Regular bulletins constitute feedback mechanisms integrating with DHIS2	The platform “Epidemic Intelligence from Open Sources” (EIOS) was recently implemented on a pilot basis, but the work needs to be extended.
Monitor, evaluate, supervise, and report information to improve surveillance and response	-Regular evaluation of information -Supervision from the health ministry -Core function indicators assess the processes and achievements	An epidemic intelligence system that encompasses early warning functions for human and animal infectious diseases is to be developed.
Electronic Integrated Disease Surveillance and Response System (e-IDSR)	The use of the health information management systems	-(DHIS2) Software is not available nationwide -Any long-term strategic plans and frameworks for e-health in countries, including health and medical IT, telemedicine, E-learning, and mHealth
System performance assessment	WHO IDSR technical guidelines enable regional comparison	Lack of systematic data collection (critical indicators) and system performance evaluation Lack of benchmarking against Joint External Evaluation (JEE) scores

## Discussion

This study provides an overview of the implementation of the IDSR in Togo, focusing on the structure and operationalization of the national strategy. Relying on secondary data limits the ability to assess potential biases in the original data collection and interpretation process.

In this study, we found that IDSR involves both indicator-based and event-based surveillance to detect priority diseases, in line with WHO recommendations. Moreover, community members play a vital role in the early detection and reporting of health events. Also, the system mandates immediate reporting of diseases with epidemic potential to national authorities. However, most of the core and support functions are partially implemented, and their full implementation faces multiple challenges and shortcomings.

Muyembe et al. conducted a systematic review to examine the key strengths, challenges, and outcomes of implementing IDSR systems in Africa in 2024. They found that the implementation levels varied across countries, with some excelling overall while others performed poorly in specific areas [15]. In the Republic of Gambia, for instance, in 2025, Indicator-Based Surveillance (IBS) was being implemented across all assessed health facilities. Still, Community-Based Surveillance (CBS) and Event-Based Surveillance (EBS) had not yet been operationalized, though stakeholders in multiple regions reported initial awareness and preparatory activities [18].

Stakeholders are oriented and trained to actively participate in detecting, reporting, responding, and monitoring human or animal health events in their intervention area. By contrast, Saleh et al. reported that there were no surveillance training plans or databases of trained surveillance personnel at the district or national levels in Zanzibar in 2021 [17]. This study showed that staff knowledge of the IDSR strategy was very low across health facilities and districts, with only 5 of 29 (17%) public primary health facility staff and 3 of 10 (30%) district surveillance staff who were able to describe the IDSR strategy perfectly [17]. The review by Muyembe et al. highlights significant deficiencies in the capacity building and training of healthcare personnel in Africa [15]

The Togolese system lacks a laboratory quality control and assurance program. Also, strengthening the links between laboratory and surveillance data needs improvement. The capacity of health facilities to diagnose or confirm priority diseases was low, as most facilities lacked the appropriate laboratory equipment and supplies needed for specimen collection or testing in Zanzibar [17]. In Ethiopia, a shortage of laboratory technicians and skilled epidemiologists has been a major barrier to effective IDSR operation in 2024 [15].

In analyzing and interpreting data, standard sheets are used to collect, compile, and analyze data along with mobile phones or computers. However, the electronic system is poorly used in rural areas. In Gambia, a significant proportion of health facilities still rely on paper-based tools, highlighting key gaps in digital infrastructure and capacity that limit the efficiency and timeliness of data submission [18]. According to this study, “data analysis was the poorest performing IDSR core function.” [18, p. 6].

To investigate suspected outbreaks and other public health events, the Togolese system relies on the rapid mobilization of the RIT, with clear, predetermined criteria, while implementing measures to prevent and contain epidemics remains the main challenge. Preparing to respond to epidemics and other public health events is hampered by insufficient domestic resources. Skilled Rapid Response Teams (RRT) in Gambia were established at both the national and regional levels, even though the country lacked a dedicated budget line for emergency preparedness and response, relying primarily on donor support [18]. In Zanzibar, Tanzania, all 10 districts reported in 2021 to have outbreak rapid response teams and budget lines specifically allocated for epidemic response, but only 60% (6/10) had developed their epidemic preparedness and response plans [17].

In responding to outbreaks and other public health events, we found that procedures for each PHEIC are coordinated at the central level, and that delays in reporting results and a lack of fluidity in collaboration are critical. At the national level, in Gambia, a National Health Emergency Steering Committee was convened on an ad hoc basis for emergency response [18].

The Electronic Integrated Disease Surveillance and Response System (e-IDSR) is underutilized and relies on health information management systems. (DHIS2) Software is not available nationwide, and there are no long-term strategic plans or frameworks for e-health in countries, including health and medical IT, telemedicine, E-learning, and mHealth. Mathenge et al. found that DHIS2 was widely used at the regional and national levels in Gambia, with gaps in data sharing with the laboratory, One Health partners, and other programs for coordination and integration [18]. It is essential to invest in this field considering its importance. Implementing the e-IDSR system “resulted in an improvement of the surveillance system in the country through the display of IDSR indicators from the weekly reports on the dashboard for real-time monitoring of disease trends and outbreaks.” [21, p. 4].

The overall challenges found in this study pertain to resource mobilization and multi-sector coordination. This situation is common in Africa. For instance, a 2024 study in Sierra Leone found that only 60% of the required funding for IDSR was obtained between 2010 and 2015, resulting in gaps in surveillance coverage and delayed outbreak responses [15]. In Tanzania, the overall IDSR implementation challenges included limited staff knowledge/training, the lack of an electronic system for reporting weekly surveillance data, and financial constraints at all levels [17]. Multiple reviews identified challenges in coordination among national health authorities, local health departments, and international partners [15,16,21]. “Fragmented efforts and a lack of synergy among stakeholders often result in duplication of activities and inefficient resource utilization” [15, p. 107].

The lack of core indicators, such as completeness and timeliness of reporting, data quality (consistency between registers and reports), coverage of reporting sites, case detection and confirmation rates for priority diseases, alongside specimen referral turnaround time, does not facilitate the system performance comparison. WHO recommends completeness and timeliness of weekly reporting at more than 80%, with data quality and coverage of reporting sites at more than 90% of health facilities [20]. The lack of systematic data collection and performance assessment is one of the main challenges hindering regional comparisons.

### Strengths and limitations

Combining policy review with stakeholder perspectives significantly strengthened the credibility of the findings and enabled us to identify gaps between operational realities and normative frameworks. The inclusion of participants at all levels (national, regional, and local) enriched the diversity of viewpoints and enabled an examination of implementation across the surveillance process. Grounding the analysis in the WHO IDSR framework ensured conceptual coherence and alignment with international health security standards.

However, several limitations must be highlighted. This was a qualitative study relying on purposive sampling. Consequently, the results are not statistically generalizable to all actors in Togo. The representation from higher technical levels was thin, which constituted an imbalance. So, contextual variations may limit their transferability. Furthermore, the lack of quantitative performance indicators for surveillance limited the ability to objectively evaluate the system. However, no substantially new themes emerged during the fourth focus group discussion, and additional data were considered unlikely to change the analytical framework. Furthermore, non-health actors and those involved in the One Health approach were underrepresented in this study. This may have negatively influenced the quality of the analysis of multisectoral coordination. In addition, the 20-day data-collection window was a significant constraint on saturation and seasonal representativeness. However, the study provides contextualized data that help strengthen surveillance implementation in Togo.

## Recommendations

Integrating IDSR modules into the initial training of health professionals and developing specific modules on diseases with epidemic potential could be exploited. It would be interesting to update existing staff’s knowledge and skills and familiarize them with innovative monitoring tools, such as EIOS. It is therefore imperative to develop training and professional development paths that meet these technological and analytical needs to strengthen epidemiological surveillance in Togo.

Improving early warning remains an essential pillar of epidemiological surveillance. With this in mind, integrating early warning functionality into the DHIS2 system, in collaboration with the Directorate of Surveillance and Emergency Response (DSUR), marks a significant step towards better notification and more effective responses to public health threats. This technological innovation will enable faster, more precise communication of health alerts, thereby strengthening the capacity to respond to epidemiological emergencies.

The commitment of the private sector and University hospital actors is decisive in strengthening integrated disease surveillance. As pillars in managing complex cases and training health personnel, university hospitals contribute significantly to the collection and transmission of epidemiological data in close collaboration with health authorities. However, deeper integration with the National Health Information System (SNIS) is imperative to overcome the shortcomings linked to the lack of reporting by many health structures.

Strengthening district-level laboratory capacity is crucial for accelerating the response to outbreaks. However, this requires significant resources and commitment.

In the context of improving epidemiological surveillance in Togo, the involvement of actors from the Ministry of Primary and Secondary Education, particularly teachers who are community leaders, could be explored as a potential avenue. Teachers can raise awareness, detect and report suspected or confirmed cases, and promote prevention and control measures. They can also contribute to social mobilization and population participation in surveillance. Collaboration with traditional medicine actors through a capacity-building mechanism in IDSR could benefit early detection and case reporting in community-based surveillance, including event-based monitoring.

The recent implementation of the pilot platform “Epidemic Intelligence from Open Sources” (EIOS) has enabled the integration of a list of media outlets in the country for disease surveillance. Work on extending this surveillance to other sources and at peripheral levels is to be done, as is practical training for EIOS users, as part of strengthening event-based surveillance.

The lack of systematical data collection and system performance assessment is one of the main challenges hampering regional comparison. Therefore, ensuring systematically data collection and the system performance assessment, conducted in Lome, is critical.

## Conclusion

This research, based on a cross-sectional qualitative study conducted in Lomé, Togo, aims to assess the level of implementation of the Integrated Disease Surveillance and Response (IDSR) Strategy and the challenges affecting its performance. We reviewed key national strategic documents and conducted focus group discussions with diverse IDSR stakeholders nationwide. The study found that IDSR is only partially implemented in Togo, with significant gaps remaining in coordination, resource mobilization, health system effectiveness, data quality and reporting, preparedness, and response capacities. Data on the completeness of reporting is unavailable even in the annual reports and there is no case detection/confirmation rates. Some recommendations aim to address key challenges across all nine core components of the IDSR strategy, thereby enhancing its performance in Togo.
