# Supplementary material for: Assessing the implementation of the disease surveillance and response strategy in Togo
Source: PLOS Glob Public Health. 2026 Sep 9;6(9):e0005698. doi: 10.1371/journal.pgph.0005698 (PMC13557351; doi:10.1371/journal.pgph.0005698)
Supplement: Supplementary file 1 [file pgph.0005698.s001.pdf]

Summary

Assessing Disease Surveillance in Togo

This study evaluates the implementation of the Integrated Disease Surveillance and Response (IDSR) strategy in Togo, identifying challenges and opportunities for improvement.

The IDSR strategy is partially implemented in Togo, with significant weaknesses in the health system.

Major challenges include inadequate coordination, limited use of digital technologies, and gaps in data quality and reporting.

The study highlights the need for reforms to strengthen surveillance and improve public health response.

The research employed qualitative methods, including document reviews and focus group discussions with key stakeholders.

Key findings on IDSR implementation includes:

- IDSR involves both indicator-based and event-based surveillance to detect priority diseases.
- Community members play a vital role in early detection and reporting of health events.
- The system mandates immediate reporting of diseases with epidemic potential to national authorities.

Minor Comments.

1. Kindly number the lines of the manuscript so we can easily refer to it when making the necessary comments
2. Kindly add the page number of the reference to the main list of references and not the main write up or manuscript
3. Kindly check your font type and size. Also do use justification if possible
4. Do not leave space to begin a paragraph. An example was seen at the result section.

Major Comments

1. Based on the breakdown of the results, can you highlight the few things that Togo is doing or not doing in subject to the WHO IDSR approved way?
2. Could you ate least compare what other countries are doing better than Togo especially within the same sub-Saharan thus if similar studies has been done.
3. Can you please make the sentence that reads “According to international trade regulations” more clearly in terms of the subject you are discussing?
4. So aside the review of the documents, I see you had a focused group discussions. How different or similar is that to reviewing the documents. It would be best if you had also included the lines of discussion and the answers that were given. Also, these people are key when it comes to surveillance hence it was highly important to share what was discussed and what the outcome of the discussion was.
