# Supplementary material for: Assessing the implementation of the disease surveillance and response strategy in Togo
Source: PLOS Glob Public Health. 2026 Sep 9;6(9):e0005698. doi: 10.1371/journal.pgph.0005698 (PMC13557351; doi:10.1371/journal.pgph.0005698)
Supplement: Supplementary file 2 [file pgph.0005698.s002.docx]

**RESPONSE TO REVIEWERS**

We sincerely thank the Editors and Reviewers for their thorough evaluation of our manuscript and for their constructive feedback and insightful comments. These suggestions and comments have substantially enhanced the clarity, methodological rigor, interpretability, and policy relevance of our work. Below, we provide a consolidated, point-by-point response to each reviewer. Aside from this Response to Reviewers, four other files have been submitted:

1. Revised Manuscript with Track Changes – showing all modifications.

2. Unmarked Version – labelled “Manuscript” – the final clean version of the revised manuscript

3. Inclusivity in global research form filled

4. Focus group discussion questionnaire (original and translated version merged)

Reviewer #1:

**Reviewer Comment**: Thank you for the opportunity to review the original research article titled “Assessing the implementation of the disease surveillance and response strategy in Togo.”

**Response:** We thank the Reviewer for his/her availability and constructive feedback

**Summary**

**Reviewer Comment:** The research article is about a cross-sectional qualitative study done in Lome, Togo to assess the level of Integrated Disease Surveillance and Response (IDSR) Strategy implementation and challenges facing its performance in Togo. The researchers reviewed key national strategic documents and conducted four focus group discussions with diverse IDSR stakeholders across Togo. Although the study found the use of IDSR in Togo, significant gaps remain in coordination, health system effectiveness, data quality and reporting, preparedness and response capacities. The study recommended addressing the key challenges across all the nine core components of the IDSR strategy to enhance IDSR Strategy performance in Togo.

**Response:** Thanks for the exact summary

**Ethics**

**Reviewer Comment:** The ethics aspect of the study was sufficiently addressed, but the authors need to include the ethics clearance reference number for clarity.

**Response:** The assessment did not require ethical clearance. It is considered a part of routine surveillance activities. However, permission was obtained from the Ministry of Health (N° 325/2024/MHPH/GS/GDRPHI/DRPP/RSD). Moreover, informed consent was obtained from the participants. Patient confidentiality and data security were upheld throughout the data collection and analysis process. Also, no personally identifiable information was collected. All data were anonymized before analysis.

**Abstract**

**Reviewer Comment:** Although there are no major gaps here, the authors should consider using the standard abstract format according to the Journal, especially with regard to the sub-section headings.

**Response:** We revised the abstract to conform to the Journal’s standard abstract format, particularly regarding the subsection headings.

**Introduction**

**Reviewer Comment:** The introduction has provided a good start with the reference guidelines from global health organizations such as World Health Organization. However, the IDSR Strategy itself is not fully described. There is an overview of each IDSR core component provided in the results section. The introduction section is the right place to integrate those descriptions into.

**Response:** The introduction section has been revised. We provided details regarding the IDSR goal, objectives, principles, core components, and functions. We removed them from the results.

**Reviewer Comment**: Additionally, the authors have not included any peer-review research papers on the IDSR Strategy, which they should have done if they want to compare their findings with other similar studies. The studies cited in the discussion section have not been highlighted in the introduction section or elsewhere until then, a gap that needs to be addressed.

**Response:** The following recent peer-reviewed research papers on the IDSR strategy have been added to the introduction and discussion. Also, the studies cited in the discussion section have been highlighted in the introduction.

C. Muyembe, E. Kazong, and R. Shitiavayi Songole, “A Systematic Review of the Integrated Disease Surveillance and Response Implementation Among African Countries Between 2010 and 2024,” Int J Health Sci Res, vol. 14, no. 10, pp. 96–112, Oct. 2024, doi: 10.52403/ijhsr.20241012.

S. D. Sasie, G. Ayano, F. Mamo, M. Azage, and M. Spigt, “Assessing the performance of the integrated disease surveillance and response systems: a systematic review of global evidence,” Public Health, vol. 231, pp. 71–79, Jun. 2024, doi: 10.1016/j.puhe.2024.03.013.

F. Saleh, J. Kitau, F. Konradsen, L. E. G. Mboera, and K. L. Schiøler, “Assessment of the core and support functions of the integrated disease surveillance and response system in Zanzibar, Tanzania,” BMC Public Health, vol. 21, no. 1, p. 748, Dec. 2021, doi: 10.1186/s12889-021-10758-0.

V. Mathenge et al., “Assessment of the National Disease Surveillance System in The Gambia, January 2025: best practices and lessons learned,” Pan Afr Med J, vol. 51, 2025, doi: 10.11604/pamj.2025.51.91.47687.

A. Abubakar, S. Idris, P. Nguku, K. Sabitu, and M. Sambo, “Assessment of integrated disease surveillance and response strategy implementation in selected Local Government Areas of Kaduna state,” Ann Nigerian Med, vol. 7, no. 1, p. 14, 2013, doi: 10.4103/0331-3131.119981.

P. Nsubuga et al., “Implementing integrated disease surveillance and response: Four African countries’ experience, 1998–2005,” Global Public Health, vol. 5, no. 4, pp. 364–380, Jul. 2010, doi: 10.1080/17441690903334943.

**Reviewer Comment**: Finally, the authors should be concise and avoid stressing points that are not clearly relevant to the study. A good example is a paragraph on the difference between communicable and infectious diseases.

**Response:** We revised the entire document, and the irrelevant parts, such as this specific paragraph, have been removed.

**Methods**

**Reviewer Comment**: The details about the study setting are clear. However, the sample size determination and sampling technique are not clear. The authors should clarify these two. For, example, how did the authors decide to select 3 participants for the first focus group discussion (FGD)? The data analysis plan is not clearly defined. What steps were taken to analyze recordings from the FGDs? The authors can also cross-check their in-text citations and format them according to the Journal's style.

**Response**: We revised the entire methods section, specifying study design, research settings, documentary analysis, and Focus Group Discussions. In the documentary analysis section, we detail the selection of documents, data extraction, and analysis. In the Focus Group Discussions, we describe participant selection, data collection, and data Analysis. We end the methods section with trustworthiness and ethical considerations. Moreover, we cross-checked the in-text citations and formatting to conform to the Journal’s style.

**Results**

**Reviewer Comment:** Generally, it would suffice to present the results without highlighting the overview of each core component in this section. The notes on core components should be moved to the introduction section. It should also be noted that the use of in-text citations in the results section is not necessary. This section should strictly present the study findings.

**Response:** We removed the overview of each core component from the results section, as the details were already in the introduction. We also remove all in-text citations in the results section, which are now strictly presenting the study findings.

**Reviewer Comment**: Additionally, the authors have not presented all the findings. While the authors have presented mostly positive findings in the core components, it is apparent in the discussion section that there are also negative findings that were not highlighted in the results section.

**Response:** We revised all nine sections of the results to present all the findings, including the positive and negative ones. Moreover, we added a sub-section dealing with the Togolese context-specific challenges falling outside the technical scope of the WHO’s IDSR core functions

**Reviewer Comment:** The authors should report all the findings regardless of whether positive or negative. Otherwise, it is not helpful to discuss findings that did not appear in the results section.

**Response:** We totally agree with the reviewer that we cannot discuss findings that did not appear in the results section. Consequently, all findings, whether positive or negative, must be presented. Now, we did so.

**Reviewer Comment:** In the methodology section, the authors mentioned the use of FGD to collect data, but in the results section, the data presentation does not reflect this. For example, there is no single quotation from a study participant throughout the results section. The authors should explain the logic behind this discrepancy.

**Response:** We revised the results section, subsection by subsection, to ensure that findings from both the documentary analysis and the FGD were presented. Consequently, quotations from the research participants are now included in the results section.

**Reviewer Comment:** Finally, the authors mentioned two tables in the results section, but none of those tables appeared in the document. The authors should consider the Journal standards on that.

**Response:** Yes, Reviewer is perfectly right! There were two tables, which had been removed. We mistakenly thought they had to be joined as separate documents. We sincerely apologize and thank the Reviewer for this special contribution. These tables are now incorporated into the manuscript, as the journal’s policies allow.

**Discussion**

**Reviewer Comment:** The discussion is not sufficient. Most of the study findings discussed here were not highlighted in the results section. It can also be noted that the authors placed the limitations of the study in the beginning of the section. Although this is not bad, it potentially distracts the reader. This should be moved to the last part of the section. The authors should also explain how they mitigated the impacts of those limitations, not just list them.

**Response:** We revised the entire discussion section, and now the study findings discussed here are highlighted in the results section. Moreover, we moved the limitations from the beginning of the discussion. We created a sub-section entitled “Strengths and Limitations” at the end of the discussion.

**Reviewer Comment:** Again, the authors need to work on reviewing more papers from similar studies to make their discussion robust. In-text citations should also be revised according to the Journal standards.

**Response:** Yes, we included 8 appropriate, similar studies in our discussion to make it robust. Six of them are mentioned above. Moreover, we revised in-text citations to comply with the journal requirements.

**Conclusion**

There are no major gaps here. However, the authors should consider making the conclusion a stand-alone part. Key findings should be highlighted and key recommendations should be explicitly stated. Additionally, recommendations should be consistent with the results and discussion sections.

**Response:** We revised the entire conclusion to make it a stand-alone section, highlighting key results and recommendations that are consistent with the results and discussion.

Reviewer #2:

**Reviewer Comment:** Kindly make available also the questions been discussed amongst the focused groups if noted down. I also did not see the tables attached to the manuscript as the annex document.

**Response:** Focus group discussion questionnaire (original and translated versions merged) has been joined. Also, we incorporated the two tables in the manuscript as the journal policy allows.

**Reviewer Comment:** Aside that, this document was well written in simple language for everyone to understand. Kudos.

**Response:** We thank the Reviewer for his/her constrictive feedback

Reviewer #3:

**Reviewer Comment:** What type of qualitative analysis was applied to the collected data, and how was this analysis conducted (analytical approach adopted, coding steps, categorization, and interpretation)?

**Response:** We revised the entire methods section, specifying study design, research settings, documentary analysis, and Focus Group Discussions. In the documentary analysis section, we detail the selection of documents, data extraction, and analysis. In the Focus Group Discussions, we describe participant selection, data collection, and data Analysis.

**Reviewer Comment**: It is recommended to explicitly specify the type of analysis applied to the qualitative data. To this end, a qualitative content or thematic analysis should be described in detail, including the steps of coding, categorization, and data interpretation. This clarification will help strengthen methodological rigor, analytical transparency, and the credibility of the study’s findings.

**Response:** In the documentary analysis section, we detail the selection of documents, data extraction, and analysis. In the Focus Group Discussions, we describe participant selection, data collection, and data Analysis. A qualitative content and thematic analysis have been described in detail, including the steps of coding, categorization, and data interpretation.
