# Supplementary material for: Assessing the implementation of the disease surveillance and response strategy in Togo
Source: PLOS Glob Public Health. 2026 Sep 9;6(9):e0005698. doi: 10.1371/journal.pgph.0005698 (PMC13557351; doi:10.1371/journal.pgph.0005698)
Supplement: Supplementary file 3 [file pgph.0005698.s003.docx]

**RESPONSE TO REVIEWERS**

We sincerely thank the Editors and Reviewers for their thorough evaluation of our manuscript and for their constructive feedback and insightful comments. These suggestions and comments have substantially enhanced the clarity, methodological rigor, interpretability, and policy relevance of our work. Below, we provide a consolidated, point-by-point response to each reviewer. Aside from this Response to Reviewers, two other files have been submitted:

1. Revised Manuscript with Track Changes – showing all modifications.

2. Unmarked Version – labeled “Manuscript (Revised 2 version)” – the final clean version of the revised manuscript

Reviewer #1:

**Reviewer Comment**: I want to thank the authors for holistically working on the reviewers’ comments. They have done a great job in revising the paper based on the suggestions made and issues identified by the reviewers.

**Response:** We have highly appreciated your suggestions.

**Reviewer Comment:** The authors have addressed the key issues I brought up in the recent review. The missing tables have been added; all sections have been revised and made clearer according to the Journal’s standards.

**Response:** We thank the Reviewer for his vigilance, which has significantly improved the quality of this work.

**Reviewer Comment:** There is still more room for improvement, though. In the results section, the authors have succinctly summarized the negative findings. However, there is a need to review the positive findings and specifically rewrite them to reflect the participants’ language. The authors can follow the language they used in presenting the negative findings: more participants’ quotes and paraphrases.

**Response:** All the result sections have been revised, and the positive findings have been rewritten to reflect the participants’ language, using quotations and paraphrases.

**Reviewer Comment:** Additionally, the authors should review the Journal’s citation style and adhere to it throughout the paper. This should be specifically done for the in-text citations. It would also be helpful to review the abbreviations, spelling, and grammar throughout the manuscript and make corrections wherever necessary.

**Response:** We revised in-text citations, primarily for multiple-source quotations. Previously, we used Zotero. We wonder if the version used was outdated. Moreover, we revised the abbreviations, spelling, and grammar throughout the manuscript and made corrections wherever necessary.

**Reviewer Comment:** Otherwise, I truly appreciate the authors for the efforts and commitment to improve the paper to meet our publication standards.

**Response:** We thank the Reviewer for his/her meaningful input.

Reviewer #2:

**Reviewer Comment:** 1. The comments has been well addressed, Thank you.

**Response:** We thank the Reviewer for his/her meaningful input.

**Reviewer Comment:** 2. I was wondering why the lines of the manuscript was not numbered? it makes it quite difficult to refer.

**Response**: We have now numbered the lines.

**Reviewer Comment:** 3. When it comes to disease surveillance, i believe it comes with cost relating to the tool or approach been used. I was wondering why the cost associated to disease surveillance was not considered? Probably it may raise some concerns that could help better surveillance response in that regard.

**Response:** We agree with the Reviewer that considering the costs associated with disease surveillance may raise concerns that could help improve surveillance response. Almost all challenges identified in this study are linked to insufficient system funding. That is, the main causes of the inappropriate implementation of IDSR are financial availability, sustainability, and equity. However, incorporating cost considerations is outside the scope of this study, which focuses on implementation rather than etiology.

**Reviewer Comment:** 4. 4. So aside the review of the documents, I see you had a focused group discussion. How different or similar is that to reviewing the documents. It would be best if you had also included the lines of discussion and the answers that were given. Also, these people are key when it comes to surveillance hence it was highly important to share what was discussed and what the outcome of the discussion was

**Response:** In our previous revised version, we included focus group discussion questionnaires in both English and French. In this study, we strategically designed the methodology combining documentary analysis and focus group discussion. Documentary analysis highlights the theoretical aspects of IDSR, which is intended. Simultaneously, focus group discussions aim to understand what is actually done and the practical aspects of IDSR.

Reviewer #4:

**Reviewer Comment:** The main question of this study is how far has gone the implementation of IDSR in Togo? I doubted that authors answered completely the question because the results presented challenges they found in the implementation but there is no clear metric or standard for comparing the results. It would be good that in a second version of this paper they said something about How much have been advanced and what remains to be done.

**Response:** We thank the Reviewer for this critical input. We revised the results sections, incorporating metric analysis. Poor system performance data collection is presented to demonstrate that the implementation is incomplete. The lack of systematic data collection and system performance assessment is one of the main challenges hampering regional comparison.

**Reviewer Comment**: Authors used documentary review for a period of 15 years. Documents were obtained from public sources at the MOH. Is there any chance that there are relevant documents that do not appear to the public? Was there any effort to identify that gap?

**Response:** Yes, we conducted a documentary review over a 15-year period. Most documents were obtained from public sources at the MoH. As we knew that there was a chance that some relevant documents would not appear publicly, we met with some critical stakeholders at the MoH after obtaining permission. We discussed with these stakeholders and obtained some documents, including confidential ones.

**Reviewer Comment**: Was a software used to code the content analysis? Or AI? If coding was manually performed, was it conducted by only one person or were there different persons involved in coding process? If there were several people involved in coding, was there any attempt to train the people.

**Response:** Coding was performed manually in Excel by two researchers after a brief training session. Moreover, we used Grammarly to reduce language spelling mistakes and Zotero to manage references.

**Reviewer Comment**: Which was the main question behind the content analysis? Given that researchers analysed different types of documents it would be useful for readers to know which questions guided content analysis for every specific batch of papers. For instance, the content searched for in outbreaks reports is surely different that the points looked for in surveillance protocols or other surveillance documents.

**Response**: The content analysis intended to grasp the theoretical and practical aspects of the implementation of IDSR in Togo and to confront them. Documents such as surveillance protocols, IDSR guidelines, and procedures were essential for a better understanding of theoretical elements. By contrast, documents such as annual reports and Joint External Evaluation (JEE) reports were critical for assessing practical aspects. We added these details in the methodology section.

**Reviewer Comment**: Results showed mainly the opinion of participants in focus group but it is not clear what patterns of responses arise from the documents that were reviewed. Some descriptions of components of the surveillance system are too long because author describe how they should function but I thing they need to focus only on the specific findings from the focus group or from the content analysis of documents.

**Response**: Results from focus group discussions and documentary analysis have been mixed, but they have helped better portray the theoretical and practical aspects. To better confront theoretical aspects of documentary analysis with practical elements from focus group discussions, some details are important. Descriptions of components of the surveillance system, which seem overly long, are based on documentary analysis.

**Reviewer Comment**: There are some points of the performance of the surveillance system where more accurate information can be obtained from documents than from interviews. It is the case of opportunity and completeness in case report and outbreak investigation where documentary review may stablish more reliably how opportune was the response to an outbreak or how is the compliance with the recommendations for case report.

**Response**: We entirely agree with the Reviewer. The accuracy of information depends on the type of measurement. This rationale guided our methodology, combining focus group discussions with documentary analysis.
