# Supplementary material for: Assessing the implementation of the disease surveillance and response strategy in Togo
Source: PLOS Glob Public Health. 2026 Sep 9;6(9):e0005698. doi: 10.1371/journal.pgph.0005698 (PMC13557351; doi:10.1371/journal.pgph.0005698)
Supplement: Supplementary file 4 [file pgph.0005698.s004.docx]

**RESPONSE TO REVIEWERS**

We sincerely thank the Editors and Reviewers for their thorough evaluation of our manuscript and for their constructive feedback and insightful comments. These suggestions and comments have substantially enhanced the clarity, methodological rigor, interpretability, and policy relevance of our work. Below, we provide a consolidated, point-by-point response to each reviewer. Aside from this Response to Reviewers, two other files have been submitted:

1. Revised Manuscript with Track Changes – showing all modifications.

2. Unmarked Version – labeled “Manuscript (Revised 4 version)” – the final clean version of the revised manuscript

**Reviewer #1:**

**Reviewer Comment:** The authors have addressed all the comments made during the last review.

**Response**: We have highly appreciated your contributions

**Reviewer Comment**: However, they should pay a closer attention to the in-text citations. The in-text citations should be consistent. I have noticed that the same citations carry page numbers at some point but none at another. There is a need to clarify or address that. Since the authors are using Zotero as the citation manager, there is a need to recheck and realign the citations.

**Response**: Thank you for this constructive feedback. We have rechecked and realigned the citations. However, direct quotations carry page numbers.

**Reviewer Comment:** Otherwise, special thanks to the authors for the effort in revising the paper.

**Response**: Thank you for all

**Reviewer #4:**

**Reviewer Comment:** No further comments

**Response**: We thank the Reviewer for his/her meaningful input.

**Reviewer #5:**

**Reviewer Comment:** Lines 71–84: The opening two paragraphs import a Codex Alimentarius/OIE trade-regulation and food safety frame that is never revisited in Methods, Results, or Discussion. Remove or condense the Codex Alimentarius/OIE trade framing; replace with a direct bridge to IDSR and IHR global health security rationale

**Response**: We condensed the Codex Alimentarius/OIE trade framing with a direct bridge to IDSR and IHR global health security rationale

**Reviewer Comment:** Table 1, items 25–26: Remove duplicate "Lymphatic filariasis" entry.

**Response**: We thank the Reviewer for his vigilance. We mistakenly duplicated “Lymphatic filariasis” instead of “Human African Trypanosomiasis (HAT)”. We have corrected.

**Reviewer Comment:** Lines 148–153: Define "Integrated Case Surveillance System (ICSS)" and its relationship to IDSR or remove from the document list if not used analytically.

**Response**: We just removed it from the document list as it was not used analytically

**Reviewer Comment**: Lines 190–193: Coding was conducted "manually, using Excel, by two researchers following a brief training." No inter-rater reliability or disagreement resolution process is described. For a qualitative study making credibility claims, the process for resolving coding discrepancies should be stated explicitly. Describe the process for resolving inter-coder disagreements; "brief training" alone does not establish coding reliability.

**Response**: We agree with the reviewer. Indeed, coding discrepancies were resolved by consensus. We have now explicitly stated.

**Reviewer Comment:** Lines 254–256, 341–342: Data on the completeness of reporting...is unavailable even in the annual reports." This is one of the study's most important empirical findings and is stated almost parenthetically. It deserves emphasis and should be flagged explicitly as a key gap finding. Elevate these key empirical findings (absence of completeness data; no case detection/confirmation rates) to explicit, emphasized conclusions rather than parenthetical observations.

**Response**: Done! We added these empirical findings in conclusion section.

**Reviewer Comment:** Lines 575–577: Replace reference [9] (influenza standards) with WHO IDSR Technical Guide [20] when citing IDSR performance thresholds.

**Response**: Done! We found this suggestion very relevant. Thank you.

**Reviewer Comment:** Lines 469–491: Integrate context-specific challenges into relevant IDSR component subsections rather than appending as a standalone section.

**Response**: Thank you for your suggestion. However, this specific subsection has been required by previous reviewers for clarity and simplicity purpose.

**Reviewer Comment**: Line 640: Replace "IDSR is used in Togo" with language consistent with the Abstract (line 59): "IDSR is only partially implemented in Togo."

**Response**: Done! Thank you for your vigilance.

**Reviewer Comment**: Lines 142–146: The 20-day data collection window (December 5–25, 2024) is a meaningful constraint on saturation and seasonal representativeness. It is not acknowledged in the Limitations (lines 588–595).

**Response**: We have now stated this constraint.

**Reviewer Comment:** Lines 204–205 vs. 28–30: Reconcile the ethics contradiction. Remove the claim that ethics clearance was not required; provide one accurate, unified ethics statement consistent with the documented IRB approval and informed consent procedures.

**Response**: Done! We thank the Reviewer for his/her meaningful input.

**Reviewer Comment**: Lines 229, 277, 453–456, 473, 479, 481, 489–490: Remove all participant percentages from Results or add a Methods paragraph explicitly describing the enumeration method for FGD responses. Qualitative thematic content analysis does not yield individual-level frequency counts from group discussions without additional procedural steps.

**Response**: Done! We removed all participant percentages from Results.

**Reviewer Comment:** Line 636–637: Correct "conducted in Lomé" to reflect the national, regional, and district scope stated in lines 142–146.

**Response**: Done! It makes sense. Thank you.

**Reviewer Comment:** Throughout Results (esp. lines 215–228, 258–281, 344–358, 440–445): Restructure each subsection to lead with empirical findings; relegate normative system descriptions to supporting context.

**Response**: We have restructured each subsection to lead with empirical findings, relegating normative system descriptions to supporting context.
