# Supplementary material for: Assessing the implementation of the disease surveillance and response strategy in Togo
Source: PLOS Glob Public Health. 2026 Sep 9;6(9):e0005698. doi: 10.1371/journal.pgph.0005698 (PMC13557351; doi:10.1371/journal.pgph.0005698)
Supplement: Supplementary file 5 [file pgph.0005698.s005.docx]

**RESPONSE TO REVIEWERS**

We sincerely thank the Editors and Reviewers for their thorough evaluation of our manuscript and for their constructive feedback and insightful comments. These suggestions and comments have substantially enhanced the clarity, methodological rigor, interpretability, and policy relevance of our work. Below, we provide a consolidated, point-by-point response to each reviewer. Aside from this Response to Reviewers, two other files have been submitted:

1. Revised Manuscript with Track Changes – showing all modifications.

2. Unmarked Version – labeled “Manuscript (Revised 5 version)” – the final clean version of the revised manuscript

Reviewer #1:

**Reviewer Comment**: (No Response)

**Response**: We thank the Reviewer for his/her previous meaningful input.

Reviewer #5:

**Reviewer Comment:** In the revised manuscript (track changes version, lines 265–306), a paragraph beginning with "IDSR is a system designed to ensure the transmission of reliable epidemiological information..." appears to be duplicated almost verbatim (lines 265–271 and again at lines 299–306). This duplication survived the revision process and must be corrected.

**Response**: We thank the author for this remark. However, in the track version, this paragraph was deleted from lines 265-271 and inserted into lines 299-306. Therefore, there was no duplication in the clean version. The previous reviewer recommended moving this paragraph from one place to another. So, it was a movement, not a duplication.

**Reviewer Comment:** While the authors added that "coding discrepancies were resolved by consensus," the analytical process still lacks sufficient detail for a credibility claim in a qualitative study. Specifically:

- How many rounds of coding occurred?

- Were inductive codes validated with participants (member checking)?

- How was theoretical saturation assessed, particularly given the acknowledged 20-day data collection window with only 21 participants across four FGDs?

The manuscript claims triangulation as a credibility strategy but does not describe how discrepancies between documentary and FGD data were handled when they arose.

**Response**: We sincerely thank the reviewer for this valuable methodological suggestion. We agree that greater transparency regarding the analytical process strengthens the credibility of qualitative research. Accordingly, we have expanded the Methods section to clarify these procedures.

We now state that the analysis involved two rounds of coding. In the first round, two researchers independently conducted deductive coding based on the WHO IDSR framework while remaining open to inductively emerging themes. In the second round, the researchers jointly reviewed and refined the coding framework, discussed coding discrepancies, merged overlapping codes, and reached consensus on the final themes. We have also clarified our validation procedures and theoretical saturation.

**Reviewer Comment:** Twenty-one participants across four FGDs is a small sample for a system assessment spanning national, regional, and district levels. The limitations section acknowledges that results are "not statistically generalizable," but does not adequately discuss whether thematic saturation was achieved. Given that the last FGD alone comprised 10 participants (community health workers and leaders), the representation from higher technical levels is thin, only six specialists from DSIERH and PHEOC combined. This imbalance should be acknowledged explicitly.

**Response**: Thanks for this analysis. We have now stated this limitation.

We added that “the representation from higher technical levels was thin, which constituted an imbalance”. We also nuanced our statement by stressing, “However, no substantially new themes emerged during the fourth focus group discussion, and additional data were considered unlikely to change the analytical framework”.

.
