# Supplementary material for: Assessing the implementation of the disease surveillance and response strategy in Togo
Source: PLOS Glob Public Health. 2026 Sep 9;6(9):e0005698. doi: 10.1371/journal.pgph.0005698 (PMC13557351; doi:10.1371/journal.pgph.0005698)
Supplement: Peer review history

# Peer review: Decision Letter 0

**DOI:** 10.1371/journal.pgph.0005698.r001

**Authors:** Kanokwan Suwannarong

29 Jan 2026

PGPH-D-25-03809

Assessing the implementation of the disease surveillance and response strategy in Togo

PLOS Global Public Health

Dear Dr. Baman,

Thank you for submitting your manuscript to PLOS Global Public Health. After careful consideration, we feel that it has merit but does not fully meet PLOS Global Public Health’s publication criteria as it currently stands. Therefore, we invite you to submit a revised version of the manuscript that addresses the points raised during the review process.

There are suggestions from the reviewers, including the need to provide number of the ethical clearance. Kindly provide it into the ethical consideration section. In addition, the discussion section should be improved by comparing with other studies.

Please submit your revised manuscript by Mar 15 2026 11:59PM. If you will need more time than this to complete your revisions, please reply to this message or contact the journal office at globalpubhealth@plos.org. When you're ready to submit your revision, log on to https://www.editorialmanager.com/pgph/ and select the 'Submissions Needing Revision' folder to locate your manuscript file.

Please include the following items when submitting your revised manuscript:

- A letter that responds to each point raised by the editor and reviewer(s). You should upload this letter as a separate file labeled 'Response to Reviewers'.
- A marked-up copy of your manuscript that highlights changes made to the original version. You should upload this as a separate file labeled 'Revised Manuscript with Track Changes'.
- An unmarked version of your revised paper without tracked changes. You should upload this as a separate file labeled 'Manuscript'.

Guidelines for resubmitting your figure files are available below the reviewer comments at the end of this letter.

We look forward to receiving your revised manuscript.

Kind regards,

Kanokwan Suwannarong, Ph.D.

Academic Editor

PLOS Global Public Health

Journal Requirements:

1.  Please include the following request in the decision letter, and ping me with follow up. “Please include a complete copy of PLOS’ questionnaire on inclusivity in global research in your revised manuscript. Our policy for research in this area aims to improve transparency in the reporting of research performed outside of researchers’ own country or community. The policy applies to researchers who have travelled to a different country to conduct research, research with Indigenous populations or their lands, and research on cultural artefacts. The questionnaire can also be requested at the journal’s discretion for any other submissions, even if these conditions are not met.  Please find more information on the policy and a link to download a blank copy of the questionnaire here: https://journals.plos.org/globalpublichealth/s/best-practices-in-research-reporting. Please upload a completed version of your questionnaire as Supporting Information when you resubmit your manuscript.

2. Please provide a complete Data Availability Statement in the submission form, ensuring you include all necessary access information or a reason for why you are unable to make your data freely accessible. If your research concerns only data provided within your submission, please write "All data are in the manuscript and/or supporting information files" as your Data Availability Statement.

If the reviewer comments include a recommendation to cite specific previously published works, please review and evaluate these publications to determine whether they are relevant and should be cited. There is no requirement to cite these works unless the editor has indicated otherwise.

Additional Editor Comments (if provided):

[Note: HTML markup is below. Please do not edit.]

Reviewers' comments:

Reviewer's Responses to Questions

**Comments to the Author**

1. Does this manuscript meet PLOS Global Public Health’s publication criteria?

Reviewer #1: Partly

Reviewer #2: Partly

Reviewer #3: Yes

**********

2. Has the statistical analysis been performed appropriately and rigorously?

Reviewer #1: No

Reviewer #2: N/A

Reviewer #3: Yes

**********

3. Have the authors made all data underlying the findings in their manuscript fully available (please refer to the Data Availability Statement at the start of the manuscript PDF file)?

The PLOS Data policy

Reviewer #1: Yes

Reviewer #2: Yes

Reviewer #3: Yes

**********

4. Is the manuscript presented in an intelligible fashion and written in standard English?

Reviewer #1: Yes

Reviewer #2: Yes

Reviewer #3: Yes

**********

Reviewer #1: Thank you for the opportunity to review the original research article titled “Assessing the implementation of the disease surveillance and response strategy in Togo.”

Summary

The research article is about a cross-sectional qualitative study done in Lome, Togo to assess the level of Integrated Disease Surveillance and Response (IDSR) Strategy implementation and challenges facing its performance in Togo. The researchers reviewed key national strategic documents and conducted four focus group discussions with diverse IDSR stakeholders across Togo. Although the study found the use of IDSR in Togo, significant gaps remain in coordination, health system effectiveness, data quality and reporting, preparedness and response capacities. The study recommended addressing the key challenges across all the nine core components of the IDSR strategy to enhance IDSR Strategy performance in Togo.

Ethics

The ethics aspect of the study was sufficiently addressed, but the authors need to include the ethics clearance reference number for clarity.

Abstract

Although there are no major gaps here, the authors should consider using the standard abstract format according to the Journal, especially with regards to the sub-section headings.

Introduction

The introduction has provided a good start with the reference guidelines from global health organizations such as World Health Organization. However, the IDSR Strategy itself is not fully described. There is an overview of each IDSR core component provided in the results section. The introduction section is the right place to integrate those descriptions into.

Additionally, the authors have not included any peer-review research papers on the IDSR Strategy, which they should have done if they want to compare their findings with other similar studies. The studies cited in the discussion section have not been highlighted in the introduction section or elsewhere until then, a gap that needs to be addressed.

Finally, the authors should be concise and avoid stressing points that are not clearly relevant to the study. A good example is a paragraph on the difference between communicable and infectious diseases.

Methods

The details about the study setting are clear. However, the sample size determination and sampling technique are not clear. The authors should clarify these two. For, example, how did the authors decide to select 3 participants for the first focus group discussion (FGD)? The data analysis plan is not clearly defined. What steps were taken to analyze recordings from the FGDs? The authors can also cross-check their in-text citations and format them according to the Journal style.

Results

Generally, it would suffice to present the results without highlighting the overview of each core component in this section. The notes on core components should be moved to the introduction section. It should also be noted that the use of in-text citations in the results section is not necessary. This section should strictly present the study findings.

Additionally, the authors have not presented all the findings. While the authors have presented mostly positive findings in the core components, it is apparent in the discussion section that there are also negative findings which were not highlighted in the results section.

The authors should report all the findings regardless of whether positive or negative. Otherwise, it is not helpful to discuss findings that did not appear in the results section.

In the methodology section, the authors mentioned the use of FGD to collect data, but in the results section, the data presentation does not reflect this. For example, there is no single quotation from a study participant throughout the results section. The authors should explain the logic behind this discrepancy.

Finally, the authors mentioned two tables in the results section, but none of those tables appeared in the document. The authors should consider the Journal standards on that.

Discussion

The discussion is not sufficient. Most of the study findings discussed here were not highlighted in the results section. It can also be noted that the authors placed the limitations of the study in the beginning of the section. Although this is not bad, it potentially distracts the reader. This should be moved to the last part of the section. The authors should also explain how they mitigated the impacts of those limitations, not just list them.

Again, the authors need to work on reviewing more papers from similar studies to make their discussion robust. In-text citations should also be revised according to the Journal standards.

Conclusion

There are no major gaps here. However, the authors should consider making the conclusion a stand-alone part. Key findings should be highlighted and key recommendations should be explicitly stated. Additionally, recommendations should be consistent with the results and discussion sections.

Reviewer #2: Kindly make available also the questions been discussed amongst the focused groups if noted down. I also did not see the tables attached to the manuscript as the annex document.

Aside that, this document was well written in simple language for everyone to understand. Kudos.

Reviewer #3: What type of qualitative analysis was applied to the collected data, and how was this analysis conducted (analytical approach adopted, coding steps, categorization, and interpretation)?

It is recommended to explicitly specify the type of analysis applied to the qualitative data. To this end, a qualitative content or thematic analysis should be described in detail, including the steps of coding, categorization, and data interpretation. This clarification will help strengthen methodological rigor, analytical transparency, and the credibility of the study’s findings.

**********

what does this mean?). If published, this will include your full peer review and any attached files.

**Do you want your identity to be public for this peer review?**  If you choose “no”, your identity will remain anonymous but your review may still be made public.

For information about this choice, including consent withdrawal, please see our Privacy Policy

Reviewer #1: No

Reviewer #2: No

Reviewer #3: **Yes:**  MORY DIAKITE

**********

[NOTE: If reviewer comments were submitted as an attachment file, they will be attached to this email and accessible via the submission site. Please log into your account, locate the manuscript record, and check for the action link "View Attachments". If this link does not appear, there are no attachment files.]

Attachment Submitted filename: PGPH-D-25-03809_comments.pdf

# Author comment: Author response to Decision Letter 1

**DOI:** 10.1371/journal.pgph.0005698.r002

16 Feb 2026

Attachment Submitted filename: RESPONSE TO REVIEWERS.docx

# Peer review: Decision Letter 1

**DOI:** 10.1371/journal.pgph.0005698.r003

**Authors:** Kanokwan Suwannarong

9 Apr 2026

PGPH-D-25-03809R1

Assessing the implementation of the disease surveillance and response strategy in Togo

PLOS Global Public Health

Dear Dr. Baman,

Thank you for submitting your manuscript to PLOS Global Public Health. After careful consideration, we feel that it has merit but does not fully meet PLOS Global Public Health’s publication criteria as it currently stands. Therefore, we invite you to submit a revised version of the manuscript that addresses the points raised during the review process.

Kindly review suggestions from Reviewer No. 2 and 4, who provided good points to improve your manuscript.

Please submit your revised manuscript by May 09 2026 11:59PM. If you will need more time than this to complete your revisions, please reply to this message or contact the journal office at globalpubhealth@plos.org. When you're ready to submit your revision, log on to https://www.editorialmanager.com/pgph/ and select the 'Submissions Needing Revision' folder to locate your manuscript file.

Please include the following items when submitting your revised manuscript:

- A letter that responds to each point raised by the editor and reviewer(s). You should upload this letter as a separate file labeled 'Response to Reviewers'.
- A marked-up copy of your manuscript that highlights changes made to the original version. You should upload this as a separate file labeled 'Revised Manuscript with Track Changes'.
- An unmarked version of your revised paper without tracked changes. You should upload this as a separate file labeled 'Manuscript'.

Guidelines for resubmitting your figure files are available below the reviewer comments at the end of this letter.

As the corresponding author, your ORCID iD is verified in the submission system and will appear in the published article. PLOS supports the use of ORCID, and we encourage all coauthors to register for an ORCID iD and use it as well. Please encourage your coauthors to verify their ORCID iD within the submission system before final acceptance, as unverified ORCID iDs will not appear in the published article. *Only*  the individual author can complete the verification step; PLOS staff *cannot*  verify ORCID iDs on behalf of authors.

We look forward to receiving your revised manuscript.

Kind regards,

Kanokwan Suwannarong, Ph.D.

Academic Editor

PLOS Global Public Health

Journal Requirements:

If the reviewer comments include a recommendation to cite specific previously published works, please review and evaluate these publications to determine whether they are relevant and should be cited. There is no requirement to cite these works unless the editor has indicated otherwise.

Please review your reference list to ensure that it is complete and correct. If you have cited papers that have been retracted, please include the rationale for doing so in the manuscript text, or remove these references and replace them with relevant current references. Any changes to the reference list should be mentioned in the rebuttal letter that accompanies your revised manuscript. If you need to cite a retracted article, indicate the article’s retracted status in the References list and also include a citation and full reference for the retraction notice.

Reviewers' comments:

Reviewer's Responses to Questions

**Comments to the Author**

Reviewer #1: (No Response)

Reviewer #2: All comments have been addressed

Reviewer #4: (No Response)

**********

publication criteria?

Reviewer #1: Yes

Reviewer #2: Yes

Reviewer #4: Partly

**********

3. Has the statistical analysis been performed appropriately and rigorously?

Reviewer #1: N/A

Reviewer #2: N/A

Reviewer #4: N/A

**********

4. Have the authors made all data underlying the findings in their manuscript fully available (please refer to the Data Availability Statement at the start of the manuscript PDF file)?

The PLOS Data policy

Reviewer #1: Yes

Reviewer #2: Yes

Reviewer #4: No

**********

5. Is the manuscript presented in an intelligible fashion and written in standard English?

Reviewer #1: Yes

Reviewer #2: Yes

Reviewer #4: Yes

**********

Reviewer #1: I want to thank the authors for holistically working on the reviewers’ comments. They have done a great job in revising the paper based on the suggestions made and issues identified by the reviewers.

The authors have addressed the key issues I brought up in the recent review. The missing tables have been added, all sections have been revised and made clearer according to the Journal’s standards.

There is still more room for improvement, though. In the results section, the authors have succinctly summarized the negative findings. However, there is a need to review the positive findings, and specifically rewrite them to reflect the participants’ language. The authors can follow the language they used in presenting the negative findings: more participants’ quotes and paraphrases.

Additionally, the authors should review the Journal’s citation style and adhere to it throughout the paper. This should be specifically done for the in-text citations. It would also be helpful to review the abbreviations, spelling and grammar throughout the manuscript and do corrections wherever necessary.

Otherwise, I truly appreciate the authors for the efforts and commitment to improve the paper to meet our publication standards.

Reviewer #2: 1. The comments has been well addressed, Thank you.

2. I was wondering why the lines of the manuscript was not numbered? it makes it quite difficult to refer.

3. When it comes to disease surveillance, i believe it comes with cost relating to the tool or approach been used. I was wondering why the cost associated to disease surveillance was not considered? Probably it may raise some concerns that could help better surveillance response in that regard.

4. 4. So aside the review of the documents, I see you had a focused group discussions. How different or similar is that to reviewing the documents. It would be best if you had also included the lines of discussion and the answers that were given. Also, these people are key when it comes to surveillance hence it was highly important to share what was discussed and what the outcome of the discussion was

Reviewer #4: The main question of this study is how far has gone the implementation of IDSR in Togo? I doubted that authors answered completely the question because the results presented challenges they found in the implementation but there is no clear metric or standard for comparing the results. It would be good that in a second version of this paper they said something about How much have been advanced and what remains to be done.

Authors used documentary review for a period of 15 years. Documents were obtained from public sources at the MOH. Is there any chance that there are relevant documents that do not appear to the public? Was there any effort to identify that gap?

Was a software used to code the content analysis? Or AI? If coding was manually performed, was it conducted by only one person or were there different persons involved in coding process? If there were several people involved in coding, was there any attempt to train the people.

Which was the main question behind the content analysis? Given that researchers analysed different types of documents it would be useful for readers to know which questions guided content analysis for every specific batch of papers. For instance, the content searched for in outbreaks reports is surely different that the points looked for in surveillance protocols or other surveillance documents.

Results showed mainly the opinion of participants in focus group but it is not clear what patterns of responses arise from the documents that were reviewed. Some descriptions of components of the surveillance system are too long because author describe how they should function but I thing they need to focus only on the specific findings from the focus group or from the content analysis of documents

There are some points of the performance of the surveillance system where more accurate information can be obtained from documents than from interviews. It is the case of opportunity and completeness in case report and outbreak investigation where documentary review may stablish more reliably how opportune was the response to an outbreak or how is the compliance with the recommendations for case report.

**********

what does this mean?). If published, this will include your full peer review and any attached files.

**Do you want your identity to be public for this peer review?**  If you choose “no”, your identity will remain anonymous but your review may still be made public.

For information about this choice, including consent withdrawal, please see our Privacy Policy

Reviewer #1: No

Reviewer #2: No

Reviewer #4: **Yes:**  Fernando de la Hoz Restrepo

**********

[NOTE: If reviewer comments were submitted as an attachment file, they will be attached to this email and accessible via the submission site. Please log into your account, locate the manuscript record, and check for the action link "View Attachments". If this link does not appear, there are no attachment files.]

# Author comment: Author response to Decision Letter 2

**DOI:** 10.1371/journal.pgph.0005698.r004

15 Apr 2026

Attachment Submitted filename: RESPONSE TO REVIEWERS (version 2).docx

# Peer review: Decision Letter 2

**DOI:** 10.1371/journal.pgph.0005698.r005

**Authors:** Kanokwan Suwannarong

1 Jun 2026

PGPH-D-25-03809R2

Assessing the implementation of the disease surveillance and response strategy in Togo

PLOS Global Public Health

Dear Dr. Baman,

Thank you for submitting your manuscript to PLOS Global Public Health. After careful consideration, we feel that it has merit but does not fully meet PLOS Global Public Health’s publication criteria as it currently stands. Therefore, we invite you to submit a revised version of the manuscript that addresses the points raised during the review process.

Kindly review the suggestions, especially from reviewer no. 5 to improve your next submission.

Please ensure that your decision is justified on PLOS Global Public Health’s publication criteria and not, for example, on novelty or perceived impact.

Please submit your revised manuscript by Jul 01 2026 11:59PM. If you will need more time than this to complete your revisions, please reply to this message or contact the journal office at globalpubhealth@plos.org. When you're ready to submit your revision, log on to https://www.editorialmanager.com/pgph/ and select the 'Submissions Needing Revision' folder to locate your manuscript file.

Please include the following items when submitting your revised manuscript:

- A letter that responds to each point raised by the editor and reviewer(s). You should upload this letter as a separate file labeled 'Response to Reviewers'.
- A marked-up copy of your manuscript that highlights changes made to the original version. You should upload this as a separate file labeled 'Revised Manuscript with Track Changes'.
- An unmarked version of your revised paper without tracked changes. You should upload this as a separate file labeled 'Manuscript'.

Guidelines for resubmitting your figure files are available below the reviewer comments at the end of this letter.

As the corresponding author, your ORCID iD is verified in the submission system and will appear in the published article. PLOS supports the use of ORCID, and we encourage all coauthors to register for an ORCID iD and use it as well. Please encourage your coauthors to verify their ORCID iD within the submission system before final acceptance, as unverified ORCID iDs will not appear in the published article. *Only*  the individual author can complete the verification step; PLOS staff *cannot*  verify ORCID iDs on behalf of authors.

We look forward to receiving your revised manuscript.

Kind regards,

Kanokwan Suwannarong, Ph.D.

Academic Editor

PLOS Global Public Health

**Journal Requirements:**

If the reviewer comments include a recommendation to cite specific previously published works, please review and evaluate these publications to determine whether they are relevant and should be cited. There is no requirement to cite these works unless the editor has indicated otherwise.

Please review your reference list to ensure that it is complete and correct. If you have cited papers that have been retracted, please include the rationale for doing so in the manuscript text, or remove these references and replace them with relevant current references. Any changes to the reference list should be mentioned in the rebuttal letter that accompanies your revised manuscript. If you need to cite a retracted article, indicate the article’s retracted status in the References list and also include a citation and full reference for the retraction notice.

[Note: HTML markup is below. Please do not edit.]

Reviewers' comments:

Reviewer's Responses to Questions

**Comments to the Author**

Reviewer #1: All comments have been addressed

Reviewer #4: All comments have been addressed

Reviewer #5: All comments have been addressed

**********

publication criteria?

Reviewer #1: Yes

Reviewer #4: Yes

Reviewer #5: Yes

**********

3. Has the statistical analysis been performed appropriately and rigorously?

Reviewer #1: N/A

Reviewer #4: N/A

Reviewer #5: No

**********

4. Have the authors made all data underlying the findings in their manuscript fully available (please refer to the Data Availability Statement at the start of the manuscript PDF file)?

The PLOS Data policy

Reviewer #1: Yes

Reviewer #4: No

Reviewer #5: Yes

**********

5. Is the manuscript presented in an intelligible fashion and written in standard English?

Reviewer #1: Yes

Reviewer #4: Yes

Reviewer #5: Yes

**********

**Reviewer #1:**  The authors have addressed all the comments made during the last review.

However, they should pay a closer attention to the in-text citations. The in-text citations should be consistent. I have noticed that the same citations carry page numbers at some point but none at another. There is a need to clarify or address that. Since the authors are using Zotero as the citation manager, there is a need to recheck and realign the citations.

Otherwise, special thanks to the authors for the effort in revising the paper.

**Reviewer #4:**  No further comments

**Reviewer #5** : Lines 71–84: The opening two paragraphs import a Codex Alimentarius/OIE trade-regulation and food safety frame that is never revisited in Methods, Results, or Discussion. Remove or condense the Codex Alimentarius/OIE trade framing; replace with a direct bridge to IDSR and IHR global health security rationale

Table 1, items 25–26: Remove duplicate "Lymphatic filariasis" entry.

Lines 148–153: Define "Integrated Case Surveillance System (ICSS)" and its relationship to IDSR or remove from the document list if not used analytically.

Lines 190–193: Coding was conducted "manually, using Excel, by two researchers following a brief training." No inter-rater reliability or disagreement resolution process is described. For a qualitative study making credibility claims, the process for resolving coding discrepancies should be stated explicitly. Describe the process for resolving inter-coder disagreements; "brief training" alone does not establish coding reliability.

Lines 254–256, 341–342: Data on the completeness of reporting...is unavailable even in the annual reports." This is one of the study's most important empirical findings and is stated almost parenthetically. It deserves emphasis and should be flagged explicitly as a key gap finding. Elevate these key empirical findings (absence of completeness data; no case detection/confirmation rates) to explicit, emphasized conclusions rather than parenthetical observations.

Lines 575–577: Replace reference [9] (influenza standards) with WHO IDSR Technical Guide [20] when citing IDSR performance thresholds.

Lines 469–491: Integrate context-specific challenges into relevant IDSR component subsections rather than appending as a standalone section.

Line 640: Replace "IDSR is used in Togo" with language consistent with the Abstract (line 59): "IDSR is only partially implemented in Togo."

Lines 142–146: The 20-day data collection window (December 5–25, 2024) is a meaningful constraint on saturation and seasonal representativeness. It is not acknowledged in the Limitations (lines 588–595).

Lines 204–205 vs. 28–30: Reconcile the ethics contradiction. Remove the claim that ethics clearance was not required; provide one accurate, unified ethics statement consistent with the documented IRB approval and informed consent procedures.

Lines 229, 277, 453–456, 473, 479, 481, 489–490: Remove all participant percentages from Results or add a Methods paragraph explicitly describing the enumeration method for FGD responses. Qualitative thematic content analysis does not yield individual-level frequency counts from group discussions without additional procedural steps.

Line 636–637: Correct "conducted in Lomé" to reflect the national, regional, and district scope stated in lines 142–146.

Throughout Results (esp. lines 215–228, 258–281, 344–358, 440–445): Restructure each subsection to lead with empirical findings; relegate normative system descriptions to supporting context

**********

what does this mean?). If published, this will include your full peer review and any attached files.

**Do you want your identity to be public for this peer review?**  If you choose “no”, your identity will remain anonymous but your review may still be made public.

For information about this choice, including consent withdrawal, please see our Privacy Policy

Reviewer #1: No

Reviewer #4: **Yes:**  Fernando de la Hoz Restrepo

Reviewer #5: No

**********

[NOTE: If reviewer comments were submitted as an attachment file, they will be attached to this email and accessible via the submission site. Please log into your account, locate the manuscript record, and check for the action link "View Attachments". If this link does not appear, there are no attachment files.]

**Figure Resubmissions:**

# Author comment: Author response to Decision Letter 3

**DOI:** 10.1371/journal.pgph.0005698.r006

5 Jun 2026

Attachment Submitted filename: RESPONSE TO REVIEWER (4).docx

# Peer review: Decision Letter 3

**DOI:** 10.1371/journal.pgph.0005698.r007

**Authors:** Kanokwan Suwannarong

29 Jun 2026

PGPH-D-25-03809R3

Assessing the implementation of the disease surveillance and response strategy in Togo

PLOS Global Public Health

Dear Dr. Baman,

Thank you for submitting your manuscript to PLOS Global Public Health. After careful consideration, we feel that it has merit but does not fully meet PLOS Global Public Health’s publication criteria as it currently stands. Therefore, we invite you to submit a revised version of the manuscript that addresses the points raised during the review process.

Please submit your revised manuscript by Jul 29 2026 11:59PM. If you will need more time than this to complete your revisions, please reply to this message or contact the journal office at globalpubhealth@plos.org. When you're ready to submit your revision, log on to https://www.editorialmanager.com/pgph/ and select the 'Submissions Needing Revision' folder to locate your manuscript file.

Please include the following items when submitting your revised manuscript:

- A letter that responds to each point raised by the editor and reviewer(s). You should upload this letter as a separate file labeled 'Response to Reviewers'.
- A marked-up copy of your manuscript that highlights changes made to the original version. You should upload this as a separate file labeled 'Revised Manuscript with Track Changes'.
- An unmarked version of your revised paper without tracked changes. You should upload this as a separate file labeled 'Manuscript'.

Guidelines for resubmitting your figure files are available below the reviewer comments at the end of this letter.

As the corresponding author, your ORCID iD is verified in the submission system and will appear in the published article. PLOS supports the use of ORCID, and we encourage all coauthors to register for an ORCID iD and use it as well. Please encourage your coauthors to verify their ORCID iD within the submission system before final acceptance, as unverified ORCID iDs will not appear in the published article. *Only*  the individual author can complete the verification step; PLOS staff *cannot*  verify ORCID iDs on behalf of authors.

We look forward to receiving your revised manuscript.

Kind regards,

Kanokwan Suwannarong, Ph.D.

Academic Editor

PLOS Global Public Health

**Journal Requirements:**

If the reviewer comments include a recommendation to cite specific previously published works, please review and evaluate these publications to determine whether they are relevant and should be cited. There is no requirement to cite these works unless the editor has indicated otherwise.

Please review your reference list to ensure that it is complete and correct. If you have cited papers that have been retracted, please include the rationale for doing so in the manuscript text, or remove these references and replace them with relevant current references. Any changes to the reference list should be mentioned in the rebuttal letter that accompanies your revised manuscript. If you need to cite a retracted article, indicate the article’s retracted status in the References list and also include a citation and full reference for the retraction notice.

**Reviewers' comments:**

Reviewer's Responses to Questions

**Comments to the Author**

Reviewer #1: All comments have been addressed

Reviewer #5: All comments have been addressed

**********

publication criteria?

Reviewer #1: (No Response)

Reviewer #5: Yes

**********

3. Has the statistical analysis been performed appropriately and rigorously?

Reviewer #1: (No Response)

Reviewer #5: Yes

**********

4. Have the authors made all data underlying the findings in their manuscript fully available (please refer to the Data Availability Statement at the start of the manuscript PDF file)?

The PLOS Data policy

Reviewer #1: (No Response)

Reviewer #5: Yes

**********

5. Is the manuscript presented in an intelligible fashion and written in standard English?

Reviewer #1: (No Response)

Reviewer #5: Yes

**********

**Reviewer #1:**  (No Response)

**Reviewer #5:**  1. In the revised manuscript (track changes version, lines 265–306), a paragraph beginning with "IDSR is a system designed to ensure the transmission of reliable epidemiological information..." appears to be duplicated almost verbatim (lines 265–271 and again at lines 299–306). This duplication survived the revision process and must be corrected.

2. While the authors added that "coding discrepancies were resolved by consensus," the analytical process still lacks sufficient detail for a credibility claim in a qualitative study. Specifically:

- How many rounds of coding occurred?

- Were inductive codes validated with participants (member checking)?

- How was theoretical saturation assessed, particularly given the acknowledged 20-day data collection window with only 21 participants across four FGDs?

The manuscript claims triangulation as a credibility strategy but does not describe how discrepancies between documentary and FGD data were handled when they arose.

3. Twenty-one participants across four FGDs is a small sample for a system assessment spanning national, regional, and district levels. The limitations section acknowledges that results are "not statistically generalizable," but does not adequately discuss whether thematic saturation was achieved. Given that the last FGD alone comprised 10 participants (community health workers and leaders), the representation from higher technical levels is thin, only six specialists from DSIERH and PHEOC combined. This imbalance should be acknowledged explicitly.

**********

what does this mean?). If published, this will include your full peer review and any attached files.

**Do you want your identity to be public for this peer review?**  If you choose “no”, your identity will remain anonymous but your review may still be made public.

For information about this choice, including consent withdrawal, please see our Privacy Policy

Reviewer #1: No

Reviewer #5: No

**********

[NOTE: If reviewer comments were submitted as an attachment file, they will be attached to this email and accessible via the submission site. Please log into your account, locate the manuscript record, and check for the action link "View Attachments". If this link does not appear, there are no attachment files.]

**Figure Resubmissions:**

# Author comment: Author response to Decision Letter 4

**DOI:** 10.1371/journal.pgph.0005698.r008

2 Jul 2026

Attachment Submitted filename: RESPONSE TO REVIEWER (5).docx

# Peer review: Decision Letter 4

**DOI:** 10.1371/journal.pgph.0005698.r009

**Authors:** Kanokwan Suwannarong

14 Jul 2026

Assessing the implementation of the disease surveillance and response strategy in Togo

PGPH-D-25-03809R4

Dear Dr Baman,

We are pleased to inform you that your manuscript 'Assessing the implementation of the disease surveillance and response strategy in Togo' has been provisionally accepted for publication in PLOS Global Public Health.

Before your manuscript can be formally accepted you will need to complete some formatting changes, which you will receive in a follow up email. A member of our team will be in touch with a set of requests.

Please note that your manuscript will not be scheduled for publication until you have made the required changes, so a swift response is appreciated.

IMPORTANT: The editorial review process is now complete. PLOS will only permit corrections to spelling, formatting or significant scientific errors from this point onwards. Requests for major changes, or any which affect the scientific understanding of your work, will cause delays to the publication date of your manuscript.

If your institution or institutions have a press office, please notify them about your upcoming paper to help maximize its impact. If they'll be preparing press materials, please inform our press team as soon as possible -- no later than 48 hours after receiving the formal acceptance. Your manuscript will remain under strict press embargo until 2 pm Eastern Time on the date of publication. For more information, please contact globalpubhealth@plos.org.

Thank you again for supporting Open Access publishing; we are looking forward to publishing your work in PLOS Global Public Health.

Best regards,

Kanokwan Suwannarong, Ph.D.

Academic Editor

PLOS Global Public Health

***********************************************************

Reviewer Comments (if any, and for reference):

Reviewer's Responses to Questions

**Comments to the Author**

Reviewer #1: All comments have been addressed

Reviewer #5: All comments have been addressed

**********

publication criteria?

Reviewer #1: Yes

Reviewer #5: Yes

**********

3. Has the statistical analysis been performed appropriately and rigorously?

Reviewer #1: N/A

Reviewer #5: Yes

**********

4. Have the authors made all data underlying the findings in their manuscript fully available (please refer to the Data Availability Statement at the start of the manuscript PDF file)?

The PLOS Data policy

Reviewer #1: Yes

Reviewer #5: Yes

**********

5. Is the manuscript presented in an intelligible fashion and written in standard English?

Reviewer #1: Yes

Reviewer #5: Yes

**********

Reviewer #1: (No Response)

Reviewer #5: No more comments

**********

what does this mean?). If published, this will include your full peer review and any attached files.

**Do you want your identity to be public for this peer review?**  If you choose “no”, your identity will remain anonymous but your review may still be made public.

For information about this choice, including consent withdrawal, please see our Privacy Policy

Reviewer #1: No

Reviewer #5: No

**********
